# Etiology-Driven Personalized Cochlear Implantation: Implications for Electrode Choice, Timing, and Outcomes

**DOI:** 10.3390/jpm16030130

**Published:** 2026-02-28

**Authors:** Chang-Hee Kim, Byung Yoon Choi

**Affiliations:** 1 Department of Otorhinolaryngology-Head and Neck Surgery, Konkuk University Medical Center, Research Institute of Medical Science, Konkuk University School of Medicine, Seoul 05030, Republic of Korea; ryomachang@gmail.com; 2 Department of Otorhinolaryngology-Head and Neck Surgery, Seoul National University College of Medicine, Seoul National University Bundang Hospital, Seongnam 13620, Republic of Korea; 3 Sensorycure Co., Ltd., Seongnam 34015, Republic of Korea; 4 Sensory Organ Research Institute, Seoul National University Medical Research Center, College of Medicine, Seoul National University, Seoul 03080, Republic of Korea

**Keywords:** hearing loss, cochlear implantation, precision medicine, personalized medicine, etiology-driven approach

## Abstract

**Background/Objectives**: A Cochlear implantation (CI) is well-established auditory rehabilitation for severe to profound sensorineural hearing loss (SNHL), yet outcomes vary widely among implantees. Even with advancements in surgical methods and device technology, CI is still commonly applied as a generally uniform procedure, with limited attention to the underlying cause of SNHL. This review aims to summarize current evidence supporting etiology-based personalization of CI and to examine how etiology influences electrode selection, implantation timing, and clinical outcomes. **Methods**: We reviewed clinical and translational studies focusing on congenital cytomegalovirus infection, genetic hearing loss, cochlear nerve deficiency, and inner-ear malformations, emphasizing how etiology influences cochlear anatomy, neural integrity, and CI outcomes. **Results**: Etiology significantly affects neural survival, cochlear anatomy, and auditory plasticity, all of which influence optimal electrode design, insertion strategy, and timing of CI. Tailoring CI approaches to specific etiologies may help explain the substantial variability in outcomes observed in both children and adults. **Conclusions**: CI should be viewed as a precision-based intervention rather than a uniform treatment. Integrating etiology into clinical decision-making is essential for advancing truly personalized CI.

## 1. Introduction

Hearing loss is one of the most prevalent sensory disorders worldwide and represents a major public health challenge with profound medical, social, and economic implications [1]. It affects individuals across the lifespan and is associated with impaired communication, reduced educational and occupational opportunities, social isolation, cognitive decline, and diminished quality of life [2,3]. Among the various forms of auditory rehabilitation, cochlear implantation (CI) stands as one of the most successful neural prostheses developed to date, restoring access to sound for individuals with severe-to-profound sensorineural hearing loss (SNHL). Over the past several decades, advances in device technology, surgical techniques, and auditory rehabilitation have led to remarkable improvements in speech perception outcomes for cochlear implantees. Nevertheless, substantial interindividual variability in CI performance persists, even among patients with apparently similar audiologic profiles.

Traditionally, decisions about CI candidacy and surgical planning have been based mainly on preoperative hearing thresholds, speech understanding scores, cogni-tive status, and basic anatomical evaluations [4,5,6], which works well for identifying, in general, who is likely to benefit from a CI. However, this strategy does not fully ac-count for the wide range of outcomes postoperatively. In reality, all CI recipients do not necessarily achieve the expected level of improvement, and some make slower or more limited progress even when the procedure was successful [7]. These patterns have led to a growing understanding that CI should be treated as a more tailored ap-proach [8,9]. As in other medical fields, CI procedures have also shifted toward per-sonalized and precision-based care. This approach aims to tailor diagnosis, prognosis, and treatment to each patient by considering their genetic, molecular, anatomical, physiological, and educational factors. Because SNHL has many different causes and often shows definitive links between specific genetic variations and clinical features, SNHL is an optimized condition for the application of personalized medicine. Recent progress in next generation sequencing (NGS), molecular testing, and high resolution imaging enables more accurate identification of the causes of SNHL, thereby facilitat-ing a more individualized approach to CI planning.

Identifying etiology is a key factor in personalized CI in SNHL, which can be caused by a wide range of genetic, infectious, developmental, degenerative, and acquired conditions, each involving different mechanisms that affect the cochlea, auditory nerve, or central auditory pathways. Genetic causes alone include hundreds of genes that influence hair cell function, synaptic signaling, spiral ganglion neuron survival, inner ear fluid homeostasis, and inner ear development [10]. Congenital infections such as cytomegalovirus can lead to progressive or fluctuating hearing loss and may also affect neural integrity and the central nervous system [11,12,13]. Likewise, inner ear malformations (IEMs), such as cochlear hypoplasia and incomplete partition (IP) defects, originate from early developmental disruptions that significantly alter cochlear structure and the interface between CI electrodes and neural tissue [14,15]. Despite this etiological diversity, CI has long been implemented using largely uniform surgical approaches and electrode selection strategies. Accumulating evidence suggests that this uniform CI strategy may contribute to unexplained outcome variability. Molecular genetic diagnosis has emerged as a powerful tool for prognostication and clinical decision making in CI candidates. Patients with identified genetic etiologies, such as pathogenic variants in *GJB2* or *SLC26A4*, often demonstrate favorable CI outcomes [16,17,18,19], while other congenital conditions, including certain forms of auditory neuropathy spectrum disorder (ANSD) or mutations affecting neural integrity, may require more individualized counseling and timing considerations [20,21,22].

## 2. Prelingual Deafness

### 2.1. Precision Diagnostic Workup in Prelingual Deafness: Why Etiology Comes First

Prelingual deafness represents a heterogeneous group of disorders arising from genetic, infectious, developmental, and acquired causes, each affecting distinct components of the auditory system. Although CI provides effective auditory rehabilitation, clinical outcomes vary substantially even among children with similar audiometric profiles. This variability reflects differences in neural substrate integrity, cochlear anatomy, and disease trajectory, underscoring the importance of identifying the underlying etiology during preoperative evaluation.

Advances in NGS and high-resolution imaging have significantly improved the ability to establish molecular and anatomical diagnoses. Genetic testing enables identification of pathogenic variants in a substantial proportion of pediatric CI candidates, allowing clinicians to distinguish between etiologies affecting primarily cochlear sensory structures and those involving synaptic or neural dysfunction [10,23]. These distinctions have direct clinical implications, as preservation of spiral ganglion neurons is a key determinant of CI responsiveness. Similarly, imaging evaluation using TBCT and MRI enables detection of inner ear malformations, cochlear nerve deficiency, and structural abnormalities that may influence electrode selection and surgical strategy [15,18,24,25,26,27,28,29,30,31,32].

By integrating molecular and imaging data, clinicians can better characterize the biological substrate available for electrical stimulation and tailor surgical planning accordingly. This etiologic stratification supports more accurate prognostic counseling, individualized electrode selection, and optimization of implantation timing to align with the child’s neural and developmental potential [33].

### 2.2. Genetic Diagnosis as the Key to Personalization

Genetic diagnosis provides critical insight into the biological mechanisms underlying hearing loss and the functional integrity of the auditory pathway. Etiologies affecting cochlear sensory transduction, such as GJB2-related deafness, typically preserve spiral ganglion neuron populations and are associated with favorable CI outcomes [34,35]. In contrast, disorders involving synaptic transmission or neural integrity, including OTOF-related auditory neuropathy or cochlear nerve deficiency, may present distinct challenges due to impaired neural synchrony or reduced neural substrate [36,37].

Beyond providing a definitive diagnosis, the molecular landscape offers actionable insights into the integrity of the neural substrate, which is the primary determinant of CI success. The lesioned site is no longer an abstract concept but a predictable variable based on genotype-phenotype correlations [5]. For instance, etiologies such as *GJB2*-related deafness, the most common genetic cause of prelingual SNHL, primarily affect the cochlear homeostatic environment while preserving the SGN population. This preservation typically translates to consistently superior speech perception outcomes and stable electrode mapping [6]. Conversely, genetic variants affecting the ribbon synapse or the auditory nerve itself, such as those found in *OTOF*-related ANSD or *PCDH15* mutations, present a vastly different biological challenge [7]. These conditions may exhibit a narrower “sensitive period” for neural plasticity or require specific electrode configurations to optimize temporal processing. By understanding the underlying pathophysiology, clinicians can reframe CI candidacy from a binary “yes/no” decision into a biologically tailored strategy, optimizing the timing of the intervention and the selection of the electrode array to match the patient’s unique neuro-anatomical potential [1,8].

Importantly, genetic information helps distinguish between etiologies in which cochlear implantation primarily bypasses sensory dysfunction and those in which neural limitations may constrain performance. This distinction enables clinicians to tailor intervention strategies and rehabilitation intensity according to the patient’s biological profile.

### 2.3. Etiology-Stratified Timing: Sensitive Periods Are Not Universal

Early CI is widely associated with improved auditory and language outcomes due to developmental neuroplasticity. However, optimal timing is not uniform across all etiologies and should be interpreted within the context of disease-specific neural and developmental factors. Etiologies affecting primarily cochlear sensory structures with preserved neural integrity, such as GJB2-related deafness, often demonstrate favorable outcomes across a relatively broad implantation window [16,18,19,23,24,25,26,27,29,38]. In contrast, synaptopathies such as OTOF-related auditory neuropathy may exhibit narrower windows for optimal neural development, supporting earlier implantation to promote cortical auditory maturation [16,20,28].

In progressive or fluctuating conditions, including SLC26A4-related hearing loss or congenital cytomegalovirus infection, implantation timing may depend on longitudinal auditory trajectory rather than chronological age alone [11,12,13,16,25,27,29,38]. Residual auditory experience prior to implantation may help preserve central auditory pathways, allowing meaningful benefit even with later implantation in selected cases.

These observations suggest that implantation timing should be guided not only by age but also by etiologic diagnosis, neural substrate integrity, and disease progression. This biologically informed approach enables clinicians to balance early neural stimulation with individualized disease characteristics, supporting optimal auditory and language development (Table 1).

### 2.4. Etiology-Driven Imaging: Inner Ear Malformations and Surgical Risk Profiling

The paradigm of pediatric CI has shifted from a standardized surgical procedure to a high precision intervention where high resolution imaging acts as the bridge between molecular etiology and surgical execution [39,40,41,42,43]. While genomic sequencing uncovers the primary biological cause of deafness, it is the synergistic use of TBCT and MRI that delineates the physical and neural landscape necessary for actionable surgical planning [44]. In the setting of IEMs, including EVA, IP I–III, cochlear hypoplasia, and common cavity, imaging extends well beyond diagnostic labeling. It enables individualized risk stratification by identifying anatomic constraints and intraoperative hazards that cannot be reliably inferred from audiometric thresholds or genetic findings alone [45].

Imaging-based stratification is particularly critical in malformations involving compromised modiolar architecture. A canonical example is IP-III, an X-linked anomaly frequently associated with *POU3F4* variants [46]. Radiographically, IP-III is characterized by the absence of the bony modiolus and an abnormally wide communication between the lateral fundus of the internal auditory canal (IAC) and the basal turn of the cochlea. These imaging hallmarks carry direct surgical implications. They predict a high likelihood of “CSF gusher” during cochleostomy and increase the risk of electrode misdirection into the IAC. Consequently, preoperative imaging can dictate a modified operative strategy, including a carefully controlled insertion trajectory, meticulous sealing methods, and in selected cases, consideration of electrode designs that reduce the probability of malposition while maintaining stable intracochlear placement [47].

Beyond identifying gross malformations, contemporary precision CI increasingly relies on quantitative morphometric assessment. Measurements such as cochlear duct length (CDL), basal turn diameter, and scala tympani geometry allow electrode selection to be individualized for optimal cochlear coverage and safety [48,49]. First, electrode length and design can be aligned with patient-specific CDL to maximize frequency coverage and support more physiologic tonotopic stimulation. Second, insertion-related trauma may be mitigated by selecting arrays that respect the altered luminal constraints of malformed cochleae. This consideration becomes especially relevant in cochlear hypoplasia or narrowed scalae, where slimmer lateral wall electrodes may offer a favorable balance between surgical feasibility and structural preservation [50]. Imaging-driven personalization also encompasses evaluation of neural integrity. Identification of CND and assessment of the bony cochlear nerve canal are central prognostic biomarkers, often best characterized using high-resolution MRI sequences such as 3D-CISS or FIESTA [51,52]. In cases of severe cochlear nerve hypoplasia or aplasia, imaging may prompt a strategic shift from CI toward ABI. Moreover, imaging can reveal concomitant central nervous system abnormalities, commonly encountered in congenital cytomegalovirus (cCMV) infection or syndromic etiologies, which may impose a ceiling on auditory and language outcomes. Incorporating these findings into preoperative counseling supports more realistic expectation setting and strengthens shared decision making.

By integrating imaging biomarkers with molecular diagnosis, pediatric CI can be reframed within a precision framework in which surgical planning is tailored not only to anatomical feasibility, but also to neural and biological potential. This multidimensional profiling reduces preventable complications, refines electrode strategy, and grounds operative decisions in the individualized neuroanatomical reality of each patient.

### 2.5. Personalized Electrode Selection in Children: Type, Length, and Modiolar Strategy

Electrode array selection in pediatric CI has moved beyond a uniform, “one-size-fits-all” approach toward a precision strategy informed by cochlear morphology and disease etiology. A central component of this decision making is the choice between lateral wall (LW) and perimodiolar (PM) electrode arrays. LW electrodes are typically slim and flexible, designed to follow the outer curvature of the scala tympani, which may reduce mechanical stress on intracochlear microstructures and help preserve the cochlear environment [50]. This consideration becomes particularly relevant in children with residual hearing or in etiologies such as *SLC26A4*-related EVA, where minimizing intracochlear disturbance may contribute to long-term stability. In contrast, PM arrays are pre-curved to achieve a closer modiolar position, thereby reducing the distance between stimulating contacts and SGNs. Such proximity may lower stimulation thresholds and decrease channel interaction, potentially benefiting children who require more focused neural activation due to reduced neural synchrony or altered neural substrate [53,54]. Recent studies reported that residual hearing can be well preserved by using PM arrays [55,56,57,58].

In parallel, inter-individual variability in cochlear size, most notably CDL, supports a personalized approach to electrode length. In children, an array that is too short may compromise frequency coverage, whereas an excessively long insertion may increase the risk of apical trauma, with potential consequences for auditory access and language development. Increasing evidence suggests that preoperative imaging-based CDL estimation can help guide array selection toward an optimal angular insertion depth (AID) that balances speech-relevant tonotopic coverage with structural safety [48,59]. This approach is especially important in IEMs such as IP, where altered internal architecture, including absent or dysplastic modiolus and interscalar septa, may increase susceptibility to kinking, malposition, or unstable intracochlear placement if electrode stiffness or length is poorly matched to the malformed cochlea [44,60].

The “modiolar strategy” also extends to situations in which the electrode–neural interface is atypical. In children with CND, the theoretical advantages of PM arrays may be less consistent because the primary target neural population is reduced. Consequently, electrode choice remains heterogeneous across centers: some favor higher contact LW arrays to provide broader current spread and maximize recruitment of any remaining neural elements, whereas others prefer PM arrays to deliver a more concentrated stimulus to limited neural fibers [52]. Ultimately, optimal electrode type and length should be determined through integration of cochlear anatomy, etiology-specific neural vulnerability, and long-term considerations, including the potential for future regenerative therapies, which may prioritize structural preservation. Within this precision framework, tailoring electrode design may also help reduce complications such as tip fold-over or scalar translocation, which occur more frequently in malformed cochleae and can compromise functional outcomes (Table 2).

### 2.6. Surgical Strategy Tailored to Etiology

In children with *GJB2*-related deafness or *SLC26A4*-associated EVA, the primary surgical objective is often to preserve intracochlear structures and maintain a stable biological environment. Such preservation is increasingly emphasized not only to support long-term neural integrity but also to keep open the possibility of future biological or regenerative interventions. In this context, “soft-surgery” principles are commonly adopted, favoring a round window approach over conventional cochleostomy to reduce intracochlear trauma and avoid abrupt perturbations in the endolymphatic milieu [61,62]. Adjunct measures, including the use of lubricants such as hyaluronic acid and slow, controlled electrode insertion, may further limit inflammatory responses and subsequent fibrosis within the scala tympani, thereby promoting a more favorable electrode–tissue interface [63]. Intraoperative topical dexamethasone administration was performed to reduce cochlear inflammation and facilitate hearing preservation. Intraoperative topical dexamethasone administration may reduce cochlear inflammation and facilitate hearing preservation [64].

By contrast, children with IEMs or post-meningitic cochlear changes often require more extensive, etiology-specific modifications to the standard surgical workflow. For example, IP-III carries a well-recognized risk of CSF gusher upon cochlear entry, mandating meticulous preoperative planning and immediate intraoperative containment [65]. Practical strategies include rapid sealing of the cochlear opening with periosteum or fibrin-based materials once the array is inserted, and in selected cases, consideration of specialized electrodes designed to assist in sealing and stabilization, thereby reducing the risk of postoperative CSF leak or and meningitis [66,67,68]. Similarly, in labyrinthitis ossificans, where cochlear patency is compromised, surgeons may employ drill-out techniques or split-array strategies to bypass ossified segments and deliver stimulation as close as possible to the remaining functional SGNs [69].

Surgical planning must also incorporate the neural integrity profile established during the etiology-driven diagnostic workup. In children with CND, the operative aim may shift toward maximizing the functional electrode–neural interface in the setting of a hypoplastic auditory nerve. This can influence decisions regarding insertion trajectory and array design, including potential preference for a perimodiolar configuration to reduce the electrode-to-nerve distance. However, this theoretical benefit must be weighed against the higher likelihood of insertion-related trauma in anatomically fragile or malformed cochleae [58,70,71,72]. Ultimately, a tailored strategy that integrates access route selection, fluid environment management, and etiology-specific electrode placement can reduce avoidable intraoperative complications and help establish a stable, high-fidelity connection between the implant and the developing central auditory system [73].

### 2.7. Intraoperative and Early Postoperative Personalization

The intraoperative phase of CI represents the first critical opportunity to confirm whether an etiology-driven surgical plan aligns with the patient’s real-time physiological and anatomical realities. Objective intraoperative measures—most notably neural response telemetry (NRT) and electrically evoked compound action potentials (ECAPs)—provide immediate feedback on the electrode–neural interface. These tools are particularly valuable in children with ANSD or synaptopathies such as *OTOF*-related hearing loss, where the clinical challenge lies in differentiating peripheral neural integrity from impaired synaptic transmission. In such contexts, intraoperative ECAP/NRT profiles can support real-time decision making regarding array positioning and stimulation capture, helping clinicians maximize the likelihood of effective neural synchronization [74]. In parallel, intraoperative impedance monitoring offers a practical safeguard against early technical issues, including air bubbles, incomplete insertion, or subtle electrode faults, which can be especially difficult to detect in malformed cochleae with distorted landmarks.

Intraoperative imaging has further expanded the precision framework by allowing immediate anatomical validation of electrode trajectory and intracochlear position. “Imaging-on-demand” approaches using mobile C-arm fluoroscopy or intraoperative 3D systems such as the O-arm have become particularly relevant in complex IEMs, including IP-III, where electrode misdirection into the IAC remains a recognized risk. By confirming the array location before wound closure, surgeons can promptly address complications such as tip fold-over, kinking, or malposition, thereby reducing the likelihood of early device underperformance and potentially avoiding revision surgery. Importantly, ensuring appropriate intracochlear placement at the initial surgery may help secure high-quality auditory input from the earliest stages of postoperative rehabilitation. The Cochlear™ Nucleus^®^ SmartNav System, a wireless intraoperative measurement tool using electrode voltage telemetry (EVT) was introduced in 2022. This system employs a sterile-dressed processor connected via Bluetooth to an Apple iPad, and conducts a placement check of electrode arrays using a proprietary trans-impedance matrix (TIM) algorithm without exposure of radiation [58].

Early postoperative personalization then shifts from anatomical confirmation to electrophysiologic and behavioral optimization during activation and mapping. Increasingly, molecular etiology is being considered alongside conventional fitting parameters to guide individualized programming strategies. For example, children with *GJB2*-related deafness often demonstrate favorable neural survival, and conventional high-rate stimulation strategies may be sufficient or advantageous. In contrast, children with CND may require alternative parameter sets—such as wider pulse widths or lower stimulation rates—to effectively recruit a limited neural population while maintaining comfort and minimizing non-auditory stimulation [75]. Through this bio-integrated approach, device activation becomes more than a standardized “turning on” process; it evolves into a personalized calibration of stimulation physiology that aims to match the individual child’s neural capacity and developmental potential.

### 2.8. Rehabilitation and Outcome Metrics in Prelingual Deafness

The success of CI in prelingual deafness is increasingly evaluated through a multidimensional framework that integrates standardized language milestones with etiology-informed expectations. Post-implant rehabilitation is not a uniform process; rather, it is a personalized, longitudinal trajectory shaped by both the timing of intervention and the underlying biological cause of hearing loss. In children with relatively favorable profiles—such as those with *GJB2*- or early-identified *SLC26A4*-associated hearing loss—outcomes are often assessed using conventional speech and auditory performance metrics, including the Categories of Auditory Performance (CAP) and the Speech Intelligibility Rating (SIR) scale. For these children, rehabilitation strategies typically emphasize higher level auditory verbal therapy (AVT) aimed at maximizing spoken language development and supporting successful integration into mainstream educational environments, reflecting the strong capacity of their auditory pathways to benefit from consistent, high-quality electrical stimulation.

In contrast, children with more complex etiologies, such as cCMV or CND, often show outcome trajectories that are not adequately captured by traditional speech perception scores alone. In these populations, rehabilitation must be adapted to account for limited neural recruitment, broader neurodevelopmental vulnerability, or concomitant cognitive delays. Accordingly, functional measures such as the Meaningful Auditory Integration Scale (MAIS) and the LittlEARS Auditory Questionnaire can provide valuable insight into incremental real world gains, even when standardized language scores remain below those of age-matched peers [76,77]. For these children, “success” may be better defined by improvements in environmental sound awareness, communication participation, and family-reported benefit, highlighting the importance of incorporating quality-of-life (QoL) indicators and total communication strategies alongside conventional linguistic endpoints.

## 3. Postlingual Deafness

### 3.1. Etiology-Driven Personalization in Postlingual CI: Beyond Audiograms

In postlingual deafness, etiology-driven personalization is increasingly reshaping CI practice, moving beyond the traditional reliance on pure-tone audiometry as the primary determinant of candidacy and prognosis. Although the audiogram provides an essential quantification of hearing thresholds, it offers limited insight into the biological state of the SGNs and the integrity of central auditory pathways—factors that often determine speech perception outcomes after implantation. Growing evidence suggests that the underlying cause of adult-onset hearing loss, ranging from genetic entities such as DFNA9 (*COCH* gene variant) to acquired or systemic conditions including otosclerosis and Ménière’s disease, meaningfully alters the neural and cochlear substrate on which the implant must operate, contributing to substantial heterogeneity in postoperative performance [38]. For instance, otosclerosis may generate distinctive electrode impedance patterns through aberrant bone remodeling, potentially necessitating stimulation strategies that differ from those used in age-related hearing loss.

Importantly, the integration of molecular diagnostics and advanced imaging enables a more refined risk–benefit assessment that cannot be captured by threshold testing alone. In clinical scenarios such as sudden sensorineural hearing loss (SSNHL) or vestibular schwannoma (VS), outcomes are frequently driven more by duration of deafness and auditory nerve health than by the degree of residual hearing measured preoperatively [78,79]. Within an etiology-driven framework, these biomarkers become central for tailoring surgical timing and for setting realistic expectations. As an example, in patients harboring mitochondrial variants associated with progressive auditory decline, earlier implantation may be considered even when audiometric candidacy appears borderline, with the goal of supporting cortical auditory engagement before significant neural deprivation or degeneration occurs [80,81,82]. This approach aligns the intervention not only with current thresholds, but also with the anticipated biological trajectory of the disease.

Personalization in the postlingual population also extends to postoperative programming and signal processing strategies informed by the presumed site of lesion. Differentiating whether the dominant pathology lies at the level of hair cells, synapses, or auditory nerve fibers can guide individualized decisions regarding frequency allocation, stimulation rate, and channel interaction management. In older adults with presbycusis, where central auditory processing limitations and cognitive listening effort play important roles, a tailored mapping may emphasize clarity and temporal cue delivery while minimizing unnecessary channel overlap, thereby reducing cognitive load during everyday speech understanding. By moving beyond the audiogram and incorporating etiology-specific neurobiological context, clinicians may reduce outcome variability and provide a more predictable and patient-centered path to auditory rehabilitation.

### 3.2. Genetic and Molecular Factors in Adult/Postlingual Hearing Loss

The genetic landscape of adult or postlingual hearing loss differs substantially from the predominantly monogenic patterns typically encountered in pediatric deafness and is more often characterized by late-onset, progressive SNHL. In adults, genetic factors may influence not only the susceptibility to hearing decline but also the pace of progression and the long-term preservation of the biological substrate required for CI (Table 3). Pathogenic variants in genes such as *COCH* (DFNA9) and *KCNQ4* (DFNA2) can drive gradual degeneration of cochlear sensory and neural structures, and their molecular mechanisms may function as a “biological clock” that determines when functional hearing deteriorates beyond the limits of amplification. Notably, *COCH*-related disease involves abnormal extracellular matrix protein deposition within the labyrinth, which can be associated with vestibulocochlear pathology and may have implications for electrode–tissue interactions and long-term stability of the electrode–neural interface [83]. Recognizing these molecular drivers supports a shift from reactive referral patterns to proactive intervention, identifying patients who may benefit from implantation before irreversible spiral ganglion loss and prolonged auditory deprivation constrain postoperative outcomes.

Beyond single gene disorders, postlingual deafness is frequently shaped by interactions between genetic susceptibility and environmental stressors, such as chronic noise exposure and ototoxic medications. This concept of molecular vulnerability suggests that certain individuals carry “hidden” genetic risks—including mitochondrial variants or disruptions in oxidative stress and antioxidant pathways—that predispose them to accelerated age-related hearing loss. Consistent with this model, genome-wide association studies (GWAS) have identified multiple loci associated with age-related hearing loss, supporting the view that adult-onset SNHL is often a complex, polygenic trait rather than a purely monogenic disorder [84,85,86]. Accordingly, incorporating genetic and molecular information into the adult CI workup may refine prognostic counseling, help anticipate postoperative speech performance, and inform individualized strategies aimed at preserving residual hearing through atraumatic surgical techniques and etiology-informed perioperative management.

### 3.3. Imaging-Based Personalization: Cochlear Patency, Ossification, and Anatomy

In postlingual deafness, high resolution imaging serves as a critical translational interface between disease etiology and individualized surgical planning. While molecular and clinical evaluation identifies the biological cause of hearing loss, imaging defines the anatomical and neural substrate available for electrical stimulation [87]. This distinction is particularly important in acquired etiologies such as meningitis, otosclerosis, and chronic inflammatory middle ear disease, in which progressive structural remodeling of the cochlea may directly affect electrode insertion feasibility and long-term implant performance [88]. Labyrinthitis ossificans, a well-recognized sequela of meningitis and severe inner ear inflammation, can result in partial or complete obliteration of the scala tympani, thereby limiting electrode insertion depth and restricting access to viable neural elements [89]. Early identification of these changes using TBCT and temporal bone MRI, particularly heavily T2-weighted sequences, enables clinicians to assess cochlear patency, detect early fibrosis or ossification, and identify the most favorable surgical trajectory.

Imaging findings in this context provide actionable guidance for surgical strategy and electrode selection. For example, focal ossification confined to the basal turn may allow partial insertion using shorter or stiffer electrode arrays [88], whereas extensive ossification may necessitate drill-out procedures or split-array implantation to achieve effective neural stimulation [90]. Conversely, preservation of cochlear fluid spaces and normal cochlear architecture supports the use of flexible lateral wall electrodes and atraumatic insertion techniques aimed at preserving intracochlear structures [91]. Thus, imaging does not merely confirm anatomical feasibility but directly informs the balance between surgical completeness, structural preservation, and neural recruitment.

Beyond identifying pathological obstruction, quantitative morphometric analysis further enables precision electrode selection by accounting for substantial inter-individual variability in cochlear dimensions. Parameters such as cochlear duct length (CDL), basal turn diameter, and overall cochlear geometry can be measured preoperatively to guide selection of electrode length and design, thereby optimizing cochlear coverage while minimizing insertion-related trauma [55,92,93,94,95]. This approach is particularly relevant in postlingual deafness, where preservation of residual neural structures and avoidance of insertion-related injury may influence long-term speech perception outcomes.

Imaging also provides important disease-specific prognostic information. In otosclerosis, abnormal bone remodeling surrounding the cochlea may alter electrical impedance characteristics and increase the risk of facial nerve stimulation due to reduced electrical resistance in the otic capsule [96,97]. Identification of these features preoperatively allows surgeons to anticipate programming challenges and consider electrode designs or insertion strategies that optimize the electrode–neural interface. Similarly, imaging can reveal structural correlates of long-standing auditory deprivation, including cochlear narrowing, neural canal stenosis, or central auditory pathway abnormalities, which may influence outcome expectations and rehabilitation planning.

Ultimately, imaging-based personalization enables clinicians to translate etiologic and pathological information into individualized surgical strategies grounded in the patient’s actual anatomical and neural substrate. By integrating structural imaging with etiologic and functional assessment, cochlear implantation can be tailored to maximize neural recruitment, minimize surgical risk, and optimize auditory outcomes within the biological constraints imposed by each patient’s disease process.

### 3.4. Etiology-Specific Surgical Considerations

In postlingual deafness, surgical strategy must be tailored not only to anatomical feasibility but also to the underlying disease process, as different etiologies produce distinct structural and electrophysiological environments within the cochlea. These disease-specific alterations influence electrode insertion mechanics, electrode–tissue interface stability, and long-term stimulation efficiency. As a result, CI has evolved from a uniform technical procedure into a biologically informed intervention in which surgical planning is adapted to the pathophysiologic consequences of the underlying etiology.

Otosclerosis provides a representative example of etiology-driven surgical adaptation [98]. This disorder is characterized by abnormal bone remodeling within the otic capsule, which may extend to the cochlear endosteum and alter the electrochemical environment of the inner ear. These changes can increase electrical impedance, disrupt current distribution, and elevate the risk of unintended facial nerve stimulation due to reduced electrical resistance between the cochlea and adjacent neural structures [89]. In this context, electrode selection becomes particularly important. PM electrode arrays may offer theoretical advantages by positioning stimulating contacts closer to the spiral ganglion neurons while directing current away from the pathologically altered lateral cochlear wall [99]. Alternatively, electrode arrays with enhanced insulation or modified contact spacing may help reduce current spread and improve stimulation specificity [100]. In advanced otosclerosis, structural changes may further compromise electrode insertion. Progressive bone remodeling can narrow or partially obstruct the scala tympani, increasing mechanical resistance during insertion and elevating the risk of scalar translocation or incomplete insertion [98]. Preoperative imaging plays a critical role in identifying these structural constraints and guiding surgical strategies. In cases with significant luminal narrowing, surgeons may employ modified insertion techniques, including careful trajectory adjustment, partial drill-out of obstructive bone, or the use of thinner, stiffer electrode arrays capable of traversing narrowed segments while maintaining intracochlear positioning [98]. These adaptations aim to maximize cochlear coverage and neural recruitment while minimizing insertion-related trauma.

Similar principles apply to other etiologies associated with acquired cochlear structural changes. In patients with labyrinthitis ossificans following meningitis, the extent and distribution of ossification determine whether standard electrode insertion is feasible or whether alternative strategies, such as partial insertion or split-array implantation, are required to stimulate residual neural populations [101]. Conversely, in patients with relatively preserved cochlear architecture, including many forms of age-related or genetically mediated hearing loss, atraumatic insertion techniques and flexible lateral wall electrodes may be preferred to preserve remaining intracochlear structures and maintain long-term neural interface stability [102].

Ultimately, etiology-specific surgical planning enables clinicians to align electrode design, insertion technique, and operative strategy with the biological and structural realities of the individual cochlea in postlingual deafness. By incorporating etiologic, anatomical, and functional information into surgical decision-making, CI can be optimized to improve neural recruitment, reduce intraoperative complications, and enhance long-term auditory outcomes. This biologically informed surgical framework represents a key component of precision CI in the postlingual population.

### 3.5. Postoperative Programming as Precision Therapy

Postoperative programming represents a critical phase in CI, during which electrical stimulation parameters are tailored to the individual’s neural substrate and functional responsiveness. Rather than serving as a standardized technical step, programming should be understood as a form of precision therapy that translates etiologic, anatomical, and electrophysiological information into individualized stimulation strategies [103]. The effectiveness of electrical stimulation depends fundamentally on the integrity, distribution, and functional responsiveness of spiral ganglion neurons, which vary substantially across etiologies and disease durations.

Etiology-driven differences in neural survival and cochlear physiology directly influence optimal programming parameters. In patients with prolonged auditory deprivation or progressive genetic etiologies such as DFNA9, neural degeneration may be heterogeneous along the cochlear spiral, resulting in variable responsiveness across electrode contacts [104]. This heterogeneity may manifest as elevated stimulation thresholds, reduced neural synchrony, or inconsistent perceptual responses. Objective electrophysiologic measures, including electrically ECAPs, NRT, and impedance telemetry, provide critical insights into the functional electrode-neural interface. These measures enable clinicians to identify electrodes with suboptimal neural coupling, adjust stimulation levels accordingly, and refine channel activation patterns to maximize effective neural recruitment while minimizing unintended stimulation [105].

Beyond peripheral neural factors, central auditory processing and cognitive function also play essential roles in determining optimal programming strategies. In older adults with age-related hearing loss or long-standing deafness, central auditory pathways may exhibit reduced temporal processing capacity or diminished neural synchrony. In such cases, conventional high-rate stimulation strategies may not provide optimal perceptual benefit and may instead increase listening effort [106]. Precision programming may therefore involve adjustments such as reducing stimulation rates, widening pulse widths, modifying frequency allocation, or selectively deactivating poorly performing electrodes to enhance temporal cue representation and improve perceptual clarity [107]. These adjustments reflect the need to align electrical stimulation not only with cochlear anatomy but also with the functional processing capacity of the central auditory system.

Importantly, postoperative programming should be viewed as a dynamic and longitudinal process rather than a single fitting event. Neural responsiveness may evolve over time due to auditory stimulation, neural plasticity, and rehabilitative training. Longitudinal monitoring using objective electrophysiologic measures and behavioral performance assessments allows progressive refinement of stimulation parameters to match the patient’s evolving neural and perceptual capacity [108,109]. This adaptive programming approach is particularly relevant in etiologies associated with progressive neural degeneration or delayed neural adaptation, where initial programming settings may require subsequent optimization.

Ultimately, treating postoperative programming as a biologically informed and adaptive therapeutic intervention enables clinicians to maximize the functional efficiency of the electrode-neural interface. By integrating etiologic diagnosis, electrophysiologic measurements, and longitudinal functional outcomes, precision programming helps bridge the gap between the patient’s biological potential and real-world communication performance [110]. This paradigm shift from uniform device fitting to individualized neural stimulation represents a central component of precision CI in the postlingual population.

## 4. Machine Learning and Big Data for Outcome Prediction and Personalization

The integration of machine learning (ML) and big data analytics has the potential to enhance CI by supporting more individualized prediction and optimization strategies. Although these approaches remain largely investigational, recent studies suggest that ML models may help identify complex relationships between genetic, anatomical, and clinical variables that influence postoperative outcomes. By leveraging multimodal datasets—including genomic information, high-resolution imaging biomarkers, intraoperative electrophysiologic metrics, and longitudinal audiologic performance—ML-based frameworks may provide complementary decision-support tools to assist clinicians in patient counseling and surgical planning, rather than replacing clinical judgment [111,112]. Establishing reliable ML models for CI personalization requires standardized, multimodal data collection across the entire care continuum. Essential datasets include (1) genomic data from targeted gene panels or whole exome/genome sequencing, enabling etiologic classification and genotype–phenotype correlation [113,114]; (2) high-resolution imaging data, including quantitative cochlear morphometry, cochlear duct length, and neural integrity assessed using TBCT and MRI [115,116]; (3) intraoperative electrophysiologic metrics, such as ECAP, impedance telemetry, and insertion depth measurements [117,118]; and (4) longitudinal postoperative outcome data, including speech perception scores, auditory performance metrics, and rehabilitation progress [119,120,121]. Integration of these multimodal datasets into structured, longitudinal registries will be essential for training robust predictive models capable of guiding personalized CI strategies. However, most existing ML models have been developed using retrospective datasets from single institutions and have not yet undergone prospective validation in diverse clinical populations. As a result, their generalizability and clinical utility remain incompletely established, highlighting the need for multicenter validation and standardized outcome reporting.

Beyond preoperative prediction, ML approaches are also being explored to support intraoperative and postoperative decision making. For example, computer vision–based systems have been investigated as potential tools to assist in analyzing intraoperative imaging and electrode positioning, with the goal of improving surgical accuracy and reducing complications such as scalar translocation. Similarly, AI-assisted programming strategies are being evaluated as potential adjuncts to conventional fitting procedures. By integrating objective electrophysiologic measurements, impedance trends, and device usage data, these systems may help identify individualized stimulation parameters and improve programming efficiency [117,122,123,124]. Nevertheless, these approaches remain in early stages of development, and their clinical utility, safety, and long-term impact on auditory outcomes require further prospective investigation before routine clinical adoption can be recommended.

Importantly, the successful implementation of ML-based personalization depends not only on algorithm development but also on the quality, diversity, and standardization of underlying clinical data. Current datasets are often limited by small sample sizes, heterogeneous outcome measures, and potential institutional biases. Furthermore, integration of ML tools into routine clinical workflows presents practical, ethical, and regulatory challenges, including data privacy considerations, interpretability of algorithmic recommendations, and the need to maintain clinician oversight in decision making.

Despite these limitations, ML and big data approaches represent promising investigational tools that may complement traditional clinical assessment and enhance personalization of cochlear implantation in the future. As large-scale, multicenter datasets become available and prospective validation studies are conducted, ML-based frameworks may help improve prognostic accuracy, optimize surgical and programming strategies, and reduce outcome variability. Until such validation is achieved, however, these technologies should be viewed as emerging adjunctive tools rather than established components of routine clinical practice.

## 5. Discussion

The concept of etiology-driven personalized CI represents a critical evolution in auditory rehabilitation, shifting the focus from population-based protocols to individualized, mechanism-informed decision making. While CI has achieved remarkable success as a neural prosthesis, persistent variability in clinical outcomes highlights the limitations of traditional uniform approaches. This variability cannot be fully explained by audiometric severity, device type, or surgical technique alone, and increasingly points to underlying etiologic heterogeneity as a dominant contributor [123,125,126,127,128]. Molecular genetic diagnosis, enabled by advances in NGS, has revealed that hearing loss previously classified as idiopathic often reflects distinct genetic entities with predictable effects on cochlear structures and neural pathways [10,23,129]. For example, patients with pathogenic variants affecting hair cell integrity but preserving SGNs, such as *GJB2*-related deafness (DFNB1), consistently demonstrate robust CI outcomes across a wide range of implantation ages. In contrast, disorders involving synaptic transmission or neural integrity, such as *OTOF*-related auditory neuropathy (DFNB9), exhibit narrower sensitive periods for effective implantation and require heightened attention to timing and postoperative rehabilitation.

Similarly, etiologic stratification offers critical insights into the management of progressive and fluctuating hearing loss. In conditions such as *SLC26A4*-related EVA or cCMV, in which hearing impairment becomes aggravating over time, residual auditory experience prior to implantation may confer advantages in post-CI performance despite delayed intervention. In such cases, rigid age-based criteria may be less informative than longitudinal assessment of disease trajectory and auditory deprivation. Etiology-driven timing decisions thus challenge conventional assumptions regarding “early versus late” implantation and emphasize individualized risk–benefit assessment. Anatomic personalization, while essential, must also be interpreted through an etiologic perspective. IEMs such as IP-III illustrate how similar radiologic phenotypes can mask underlying molecular heterogeneity with important functional consequences. Recent genotype–outcome correlations in *POU3F4*-associated malformations suggest that the nature of the genetic alteration may influence auditory performance following CI, even when surgical challenges and electrode positioning appear comparable [130]. These findings underscore the inadequacy of anatomy-only frameworks and reinforce the value of integrating molecular data into surgical planning and counseling. However, it is also important to recognize that gene expression and its functional consequences are not static across the lifespan [131]. Age-dependent changes in gene expression, neural survival, and synaptic integrity may influence the responsiveness of the auditory system to electrical stimulation. For example, prolonged auditory deprivation may result in progressive spiral ganglion degeneration, even in genetic conditions primarily affecting hair cells [132]. Conversely, early implantation may preserve neural connectivity and support more favorable cortical auditory development [133]. These observations highlight that genetic etiology must be interpreted in conjunction with age and disease duration, reinforcing the importance of timely intervention to maximize neural preservation and functional outcomes.

Beyond genetics and anatomy, etiology-driven personalization has implications for electrode selection and surgical strategy. The choice between LW and PM electrodes, as well as decisions regarding electrode length and insertion depth, should be informed not only by cochlear dimensions but also by expected neural distribution and vulnerability. Disorders characterized by abnormal neural trajectories or absent modiolar structures may benefit from electrode designs prioritizing broad neural coverage and reduced insertion risk. Such considerations exemplify how etiology transforms device selection from a technical preference into a biologically informed decision. Importantly, etiology-driven personalization extends beyond the operating room. Postoperative programming, rehabilitation intensity, and outcome expectations should be aligned with disease mechanism and neural plasticity. Patients with synaptic or neural disorders may require prolonged auditory training and alternative outcome metrics beyond conventional speech perception scores. In this context, emerging computational approaches that integrate genetic, imaging, and audiologic data hold promise for refining predictive models and further advancing precision CI care [123]. Despite these advances, several challenges remain. The rarity of many genetic etiologies limits large-scale outcome studies, and the complexity of genotype–phenotype relationships complicates clinical translation. Furthermore, access to molecular diagnostics and advanced imaging remains uneven across healthcare systems. Addressing these barriers will require multidisciplinary collaboration, standardized data collection, and continued integration of precision medicine principles into routine otologic practice.

Despite the compelling rationale for etiology-driven personalization in CI, several important limitations should be acknowledged. First, much of the existing evidence is derived from retrospective studies, single-center experiences, and relatively small cohorts, particularly for rare genetic etiologies. These limitations restrict statistical power and may introduce selection bias, thereby limiting the generalizability of reported outcomes. Second, long-term outcome data remains insufficient for many genetic subtypes and complex etiologies, especially those associated with progressive neural degeneration or multisystem involvement. As a result, the durability of CI performance and the lifelong trajectory of auditory and language development in these populations are not yet fully characterized. Third, implementation of etiology-driven personalization depends on access to advanced diagnostic tools, including NGS and high-resolution imaging, which are not uniformly available across healthcare systems. This variability may contribute to disparities in care and limit the widespread adoption of precision-based approaches, particularly in resource-constrained settings. Fourth, many published outcome studies originate from high-volume tertiary referral centers with extensive surgical experience and multidisciplinary rehabilitation resources, which may lead to outcome estimates that are not fully representative of broader clinical practice. Finally, ethical, economic, and practical considerations must be carefully addressed, including the cost-effectiveness of routine genomic screening, challenges in interpreting variants of uncertain significance, and the potential psychological and counseling implications for patients and families. Addressing these limitations will require prospective multicenter studies, standardized outcome measures, equitable access to advanced diagnostics, and continued integration of genomic and clinical data into comprehensive precision medicine frameworks for CI.

## 6. Conclusions

CI should be reconceptualized as a precision intervention guided by disease etiology rather than a standardized solution for profound HL. Importantly, etiology-driven personalization provides actionable guidance for surgical decision-making rather than serving only as a prognostic descriptor. For example, in synaptopathies such as OTOF-related hearing loss, early implantation is strongly recommended to maximize auditory cortical development, as delayed intervention may result in suboptimal neural synchronization despite preserved cochlear morphology. Conversely, in progressive conditions such as SLC26A4-related hearing loss, implantation timing may be individualized based on longitudinal auditory trajectory rather than fixed age criteria, because meaningful outcomes remain achievable even with later implantation if neural integrity is preserved. Similarly, in cases of cochlear nerve deficiency, electrode selection and surgical expectations must be tailored to the reduced neural substrate, and in severe cases, alternative interventions such as auditory brainstem implantation may be considered. These examples illustrate that etiology provides clinically actionable information regarding both the optimal timing and technical approach to CI. Although etiology provides critical biological insight, surgical decision-making is ultimately determined by the anatomical and functional phenotype of the auditory system, including cochlear morphology, neural integrity, and disease progression. Etiology serves as the upstream determinant that shapes these structural and functional characteristics. Therefore, etiology-driven personalization should be understood as a biologically informed framework that integrates molecular diagnosis with anatomical and physiological assessment. This integrated approach enables clinicians to translate etiologic information into practical surgical decisions based on the patient’s actual neural and anatomical substrate. As diagnostic technologies and data-driven tools continue to evolve, embracing etiology as the cornerstone of personalized CI will be essential for maximizing the rehabilitative potential of this transformative therapy.

## Figures and Tables

**Table 1:** Etiology-stratified considerations for timing of CI in prelingual deafness.

Etiology	Disease Biology	Natural History	Timing Implication
***GJB2* (DFNB1)**	Hair cell dysfunction, neural substrate,	Typically stable severe-profound HL	Broad window: earlier generally better, but later CI can still succeed
***SLC26A4* (DFNB4)**	Progressive/fluctuating cochlear pathology	Often progressive, may retain auditory experience	Timing guided by trajectory rather than age alone; later CI may still do well
***OTOF* (DFNB9)**	Synaptopathy/auditory neuropathy spectrum disorder	Stable profound with “hidden” encoding deficit	Narrower sensitive period; earlier implantation and intensive rehabilitation emphasized
**cCMV**	Mixed cochlear + possible central involvement	Progressive + variable neurodevelopment	Timing individualized; benefit possible but outcome variability higher
***POU3F4* (DFNX2)**	Developmental malformation with abnormal modiolar/nerve anatomy	Severe/progressive; high surgical complexity	Timing must integrate surgical risk and rehabilitation, not age alone
**Cochlear nerve deficiency**	Neural hypoplasia/aplasia	Variable, often severe	Timing alone cannot overcome neural limitation; CI vs ABI counseling

HL, hearing loss; cCMV, congenital cytomegalovirus infection, ABI, auditory brainstem implant.

**Table 2:** Etiology-driven electrode selection and surgical strategy.

Etiology/Malformation	Anatomical/Physiological Challenge	Recommended Electrode Strategy	Rationale
***GJB2/SLC26A4***	Well-preserved SGNs; Risk of progressive loss or pressure changes	Slim LW	Prioritizes structural preservation and minimizes insertion trauma to protect the delicate intracochlear environment.
**Labyrinthine ossification**	Fibro-osseous obliteration of the scala tympani	Stiff/mid-length/split-array	Requires sufficient mechanical stiffness to bypass or drill through obstructions; shorter or split arrays may be necessary for partial ossification.
**IP-II**	Enlarged vestibule; slightly shorter cochlear duct	Medium-length LW or PM	Standard arrays often fit well, but length must be carefully chosen to avoid apical overcrowding in a shorter duct.
**IP-III**	Absent modiolus; CSF gusher risk; IAC communication	Form-fitting LW or custom	Avoids perimodiolar arrays that may “kink” into the IAC; favors arrays that can be easily sealed at the cochleostomy to manage high-pressure CSF.
**Cochlear hypoplasia/common cavity**	Significant structural dysmorphism; limited scalar space	Short LW or specialized straight	Avoid “tip fold-over” in restricted spaces; length is strictly limited by the total lumen available.
**Cochlear nerve deficiency**	Scarcity of SGNs	PM	Aims to place electrodes as close to the modiolus as possible to maximize recruitment of the sparse remaining neural fibers.
***OTOF***	Intact hair cells but neural asynchrony	High-density LW or PM	Focuses on high-fidelity, synchronized stimulation; preservation of hair cells (via LW) may be relevant for future gene therapies.

SGN, spiral ganglion neuron; LW, lateral wall; IP, incomplete partition; PM, perimodiolar; CSF, cerebrospinal fluid; IAC, internal auditory canal.

**Table 3:** Genetic causes of postlingual/late-onset hearing loss and CI implications.

Gene (Locus)	Phenotype/Pattern	Pathophysiology	CI Implications
***COCH* (DFNA9)**	Adult-onset (20 s–50 s); Progressive SNHL + Vestibular dysfunction.	Acidophilic mucopolysaccharide deposits in the cochlea/vestibule	May lead to increased electrical impedance; excellent outcomes if SGNs are preserved.
***KCNQ4* (DFNA2)**	Early-adult onset; High-frequency progressive loss	Dysfunction of potassium channels in outer hair cells	Excellent CI prognosis; pathology is primarily at the hair cell level, leaving SGNs intact
***WFS1* (DFNA38/6)**	Low-frequency SNHL; Slowly progressive	Endoplasmic reticulum stress in the membranous labyrinth	Favorable outcomes; low-frequency focus may benefit from Electric-Acoustic Stimulation
***POU4F3* (DFNA15)**	Late-onset (30 s); Progressive SNHL	Transcription factor defect; gradual loss of hair cells	Good outcomes due to secondary nature of SGN degeneration
***SLC26A4* (DFNB4)**	Progressive or fluctuant SNHL; EVA-associated	Ionic/fluid imbalance in the endolymph	Risk of “CSF gusher” (though less than IP III); requires soft surgery to stabilize fluctuations
**Mitochondrial (e.g., m.1555A > G, m.3243A > G)**	Late-onset; Often triggered by aminoglycosides (m.1555A > G)	Mitochondrial protein synthesis defect leading to metabolic failure	Outcomes vary by duration of deafness; generally positive if implanted before severe neural loss

SGN, spiral ganglion neuron; SNHL, sensorineural hearing loss; EVA, enlarged vestibular aqueduct; CSF, cerebrospinal fluid.

## Data Availability

No new data were created or analyzed in this study. Data sharing is not applicable to this article.

## Abbreviations

The following abbreviations are used in this manuscript:

CI	Cochlear implant
SNHL	Sensorineural hearing loss
CND	Cochlear nerve deficiency
ABI	Auditory brainstem implant
NGS	Next-generation sequencing
IEM	Inner ear malformation
IP	Incomplete partition
EVA	Enlarged vestibular aqueduct
CSF	Cerebrospinal fluid
ANSD	Auditory neuropathy spectrum disorder
SGN	Spiral ganglion neuron
IAC	Internal auditory canal
CDL	Cochlear duct length
cCMV	Congenital cytomegalovirus
LW	Lateral wall
PM	Perimodiolar
AID	Angular insertion depth
CAP	Categories of auditory performance
AVT	Auditory verbal training
MAIS	Meaningful Auditory Integration Scale
SSNHL	Sudden sensorineural hearing loss
VS	Vestibular schwannoma
ML	Machine learning
